# Secretomics reveals hormone-therapy of breast cancer may induce survival by facilitating hypercoagulation and immunomodulation in vitro

**DOI:** 10.1038/s41598-023-49755-1

**Published:** 2024-01-17

**Authors:** Tanya N. Augustine, Sindisiwe Buthelezi, Kyrtania Pather, Kutlwano R. Xulu, Stoyan Stoychev

**Affiliations:** 1 School of Anatomical Sciences, Faculty of Health Sciences, University of the Witwatersrand, Johannesburg, South Africa; 2 Department of Biosciences, Council for Scientific and Industrial Research, Pretoria, South Africa

**Keywords:** Breast cancer, Proteomics, Experimental models of disease

## Abstract

Tumour cell haematogenous dissemination is predicated on molecular changes that enhance their capacity for invasion and preparation of the pre-metastatic niche. It is increasingly evident that platelets play an essential role in this transformation. The systemic nature of signalling molecules and extravascular factors that participate in mediating platelet-tumour cell interactions led to the development of an in vitro co-culture using whole blood and breast tumour cells, allowing us to decipher the impact of hormone-therapy on tumour cells and associated changes in the plasma proteome. Using mass spectrometry, we determined dysregulation of proteins associated with maintaining an invasive tumour phenotype. Tumour changes in genes associated with EMT and survival were documented. This is postulated to be induced via tumour cell interactions with the coagulatory and immune systems. Results highlight tumour cell adaptability to both treatment and blood resulting in a pro-tumorigenic response and a hypercoagulatory state. We illustrate that the breast cancer cell secretome can be altered by hormone-therapy, subject to the tumour subphenotype and linked to platelet activation. More sophisticated co-culture systems are required to recapitulate these interactions to better understand tumorigenesis. Moreover, deeper plasma profiling, using abundant protein depleted and/or vesicle enriched strategies, will likely reveal additional secretory proteins related to tumour cell-platelet interactions.

## Introduction

The haematogenous dissemination of tumour cells is predicated on molecular and phenotypic changes in tumour cells that enhance their capacity for invasion, migration, immune evasion, and preparation of the pre-metastatic niche at secondary sites. It has become increasingly evident that platelets and platelet-derived microparticles (PMPs) play an essential role in this transformation. Interaction of these elements with tumour cells have been documented in situ within the tumour bed, where extravascular platelets interact with primary tumour cells; and within the vasculature itself, where intravascular platelets associate with circulating tumour cells^1–6^. Tumour cells can activate platelets by releasing a host of coagulation factors including ADP, tissue factor and thrombin^7,8^. In addition to promoting thrombosis, activated platelets in turn release growth factors and cytokines that firstly permit tumour cell acquisition of a more aggressive phenotype in preparation for metastasis; secondly, that guide the formation of the pre-metastatic niche; and thirdly, facilitate physical cloaking of tumour cells by platelets to prevent immune cell recognition and lysis^2,9,10^. Immune system involvement in the tumour microenvironment has long concentrated on the cellular fractions of the innate and adaptive immune systems. However, there is evidence for the impact of immunoglobulins and complement, evolutionary ancient components of the immune system, in modulating tumour progression^11,12^.

In vitro studies have shown that platelet-tumour cell interactions heighten hypercoagulatory phenomena and drive tumour processes including epithelial-mesenchymal transition (EMT), thereby enhancing the aggressive characteristics of a range of hormone-dependent cancer cell lines derived from, for example, the prostate gland, ovary and breast^8,10,13^. In breast cancer, standard hormone-therapies include Anastrozole and Tamoxifen. Anastrozole, an aromatase inhibitor, is used primarily for post-menopausal breast cancer patients preventing the conversion of testosterone into oestradiol^14^. Tamoxifen remains the gold-standard for hormone-dependent breast tumours, as a selective oestrogen-receptor modulator that prevents downstream engagement of signalling pathways involved in tumour progression^15,16^. However, while these hormone-therapies offer good clinical benefit they are still plagued by varying levels of risk of thrombotic complications, resistance to therapy, and disease recurrence that negatively affect patient outcomes^14–17^. The systemic nature of signalling molecules, as well as other intra- and extravascular factors that are involved in mediating platelet-tumour cell interactions led us to develop an in vitro co-culture model to decipher the impact of hormone-therapy on breast tumour cells and associated changes in the plasma proteome. We were thus able to ascertain changes in tumour cell gene expression associated with EMT and survival, as well as dysregulation of proteins in plasma that may function in maintaining an invasive phenotype by harnessing the power of the coagulatory and immune systems and exploiting extracellular matrices.

## Materials and methods

Ethical clearance was obtained from Human Research Ethics Committee (Medical), University of the Witwatersrand (Clearance Certificate Number M160826). Informed consent was obtained from all volunteers who donated whole blood and all research was performed in accordance with ethical guidelines.

### Cell culture and hormone-therapy

Breast cancer cell lines MCF7 (passage number 36) and T47D (passage number 29), donated by Prof R. Duarte (Dept. Internal Medicine, School of Clinical Medicine, University of the Witwatersrand) were cultured in DMEM (Dulbecco’s Modified Eagles Medium, Lonza, Walkersville, MD, USA), with 10% FBS (Fetal Bovine Serum, Gibco, Life Technologies, Johannesburg, South Africa), 0.1% P/S (Penicillin/Streptomycin, Sigma-Aldrich, St. Louis, MO, USA) and RPMI (Roswell Park Memorial Institute medium) with 0.2 units/ml bovine insulin, 10% FBS and 0.1% P/S, respectively. Cell viability was assessed using the trypan blue exclusion assay with a TC20 Automated Cell Counter (Bio-Rad, Hercules, CA, USA). For experimentation cells were seeded at 1 × 10^5^ cells per well in a 24-well plate and allowed to adhere for 24 h, followed by treatment with 1 μM Anastrozole or 2 μM Tamoxifen, as previously conducted in our lab^8,18,19^, in normal media for 24 h at 37 °C and 5% CO_2_. Normal media controls were included.

### Whole blood preparation and co-culture

Peripheral whole blood (WB) was donated by healthy female volunteers (maximum n = 6, aged between 19 and 30 years old) between days 1–10 of their menstrual cycle in which reduced levels of circulating oestrogen and progesterone present^20^. Exclusion criteria included previous history of cancer or current cancer diagnosis, smoking, autoimmune diseases or immunodeficiency, pregnancy, contraceptive use, and consumption of anti-platelet and/or anti-coagulation medication in the previous 72 h. WB was drawn by a phlebotomist (Day Ward, Charlotte Maxeke Academic Hospital, Johannesburg, South Africa) into 3.2% sodium citrate vacutainers. The effect of mechanically activated platelets was excluded by discarding the first 2 ml of blood. 200 µl of WB was co-cultured with breast cancer cells for 2.5 min at room temperature (RT), as previously established in our lab^19^. Following co-culture with WB, breast cancer cells were prepared for gene expression assays, whereas WB was prepared for scanning electron microscopy or proteomic analysis. WB, for proteomic analysis, was centrifuged at 4 °C at 1500×*g* twice, for 10 min, the top plasma layer removed and snap frozen in liquid nitrogen and stored at − 80 °C.

### Gene expression analysis

#### RNA extraction

Following co-incubation of cell lines with WB, cells were rinsed 3 times with 0.1 M PBS. Total cellular RNA was extracted using the RNeasy Mini-Kit (Qiagen, catalogue number: 74104), as per manufacturer’s recommendation. The RNA elute was stored at − 80 °C. RNA concentration and quality (260 nm/280 nm ratio) was determined using a Nanodrop 2000 spectrophotometer (ThermoFisher Scientific, Wilmington, USA). RNA integrity (RINs) was analysed using an RNA 600 Pico Kit (Agilent, Johannesburg; 5067-1513) and Bioanalyzer 2100 (Agilent Technologies). All RINs values obtained were between 8 and 10, indicating a high integrity value for RNA samples used for cDNA synthesis.

#### cDNA synthesis and qPCR

cDNA was synthesised using the High-Capacity cDNA reverse transcription kit (Applied Biosystems, catalogue number: 4368814) as per manufacturer’s guidelines. The MJ Mini Personal Thermocycler (Bio-Rad, Rosebank, South Africa) was used at the following conditions; 25 °C for 10 min, 37 °C for 120 min and 85 °C for 5 s, the resulting cDNA was stored at − 20 °C. Primers were designed on the Eurofins website (www.eurofinsgenomics.com) then validated using the oligoanalyzer tool on the IDT website (www.idtdna.com) and primer blast (www.ncbi.nlm.nih.gov) (Table 1). Gene amplification was conducted in a total reaction volume of 10 μl containing 200 nM forward and reverse primer each, 2 ng cDNA and 2 × SYBR green (Applied Biosystems, catalogue number A25742). The QuantStudio1 thermocycler (Thermo Fisher Scientific) was used at the following conditions: hold stage (50 °C for 2 min, 95 °C for 2 min), PCR stage (95 °C for 15 s, 60 °C for 15 s, 72 °C for 1 min) and melt curve stage (95 °C for 15 s, 60 °C for 1 min, 95 °C for 15 s).

**Table 1:** Primers sequences used for gene amplification with qPCR and/or ddPCR.

Gene	Primer sequence (5′–3′)	Accession number
*GAPDH *(reference gene)	**F:** TGCACCACCAACTGCTTAGC **R:** GGCATGGACTGTGGTCATGAG	NM_002046.5
*RPLO *(reference gene)	**F:** TGCAGCTGATCAAGACTGGAGACA **R:** TCCAGGAAGCGAGAATGCAGAGTT	BC_001834.2
*ACTB3*	**F:** GGCCGAGGACTTTGATTGCAC **R:** TTAGGATGGCAAGGGACTTCCTGT	NM_001101.3
***AKT1***	**F: TGTCATCGAACGCACCTTCC** **R: ACACCTCCATCTCTTCAGCC**	**NM_005163.2**
*TGFβ-1*	**F:** CTCGCCAGAGTGGTTATCTT **R:** AGTGTGTTATCCCTGCTGTC	NM_000660.7
*Vimentin*	**F:** CCT CTT CCA AAC TTT TCC TCC **R:** CGT TGA TAA CCT GTC CAT CTC	NM_003380.5
*ESR1*	**F:** TATGTGTCCAGCCACCAACC **R:** TCGGTCTTTTCGTATCCCACC	NG_011535.1
*ESR2*	**F:** CGGCAGACCACAAGCCCAAATG **R:** CGATCTTGCTTCACACCAGGGAC	NG_008493.2

Significant values are in bold.

#### Absolute quantification of *ESR1*, *ESR2 * and *ACTB3* using ddPCR

To quantify absolute concentrations of *ESR1*, *ESR2* and *ACTB3* in each sample, a reaction mixture of 20 μl was prepared containing 5 ng cDNA, forward and reverse primers (100 nM each) and ddPCR Multiplex Supermix (Bio-Rad, catalogue number: 12005909). Droplets were generated using the QX200 Droplet Generator (Bio-Rad) and transferred into a 96-well plate for amplification. The plate was loaded into the QX200 Droplet reader (Bio-Rad) which analysed each droplet per sample for the presence or absence of the target DNA. The QX200 Quantasoft software was used to quantify the levels of *ESR1*, *ESR2* and *ACTB*3 in each sample tested.

#### Data analysis

Data was managed in Microsoft Excel. For relative quantification of genes following qPCR, the mean fold change was calculated as previously described^21^. Statistical analysis was conducted using Statistica V23 software. The non-parametric Kruskal–Wallis ANOVA test and corresponding post-hoc test was conducted to determine whether gene expression, relative (qPCR) or absolute (ddPCR), significantly altered (p < 0.05) in response to hormone-therapy.

### Scanning electron microscopy

WB was aspirated and erythrocytes lysed in ammonium chloride buffer at a ratio of 1:2. The suspension was centrifuged at 200×*g* for 5 min and the pellet resuspended in 150 μl Tyrode’s buffer. 20 μl each of suspension was transferred to a glass coverslip within 24 well plates, incubated at 37 °C in 5% CO_2_ for 5 min, and washed in 0.1 M PBS on a microplate shaker for 20 min. Primary fixation was in 2.5% formaldehyde/glutaraldehyde for 15 min followed by rinsing in 0.1 M PBS and secondary fixation in 1% osmium tetroxide. Samples were rinsed further in 0.1 M PBS and dehydrated in a graded series of ethanol and hexamethyldisilazane. Samples were mounted onto aluminium stubs, carbon coated and viewed on a FEI Nova 600 Scanning Electron Microscope (Microscopy and Microanalysis Unit, University of the Witwatersrand) with acceleration voltage set at 30 kV.

### Plasma sample preparation for proteomics analysis

Stored plasma samples were thawed on ice, then diluted to 10 × with a 50 mM Tris buffer and 2% SDS. Total protein was estimated using the 2-D Quant Protein Assay Kit (GE Healthcare, Amersham, UK). For downstream processing, 20 µg was taken from each sample, transferred to a new 1.5 ml tube, reduced with 10 mM Dithiothreitol (DTT) for 30 min at 60 °C and followed by alkylation of free thiols using 40 mM Iodoacetamide (IAA) incubated in the dark at RT for 30 min. Excess IAA was quenched with a further addition of DTT to a final concentration of 10 mM.

#### Digest preparation

On-bead protein capture, clean-up and digestion was done using the automated KingFisher™ Duo magnetic liquid handling station (Thermo Fisher Scientific, Massachusetts, USA) and MagReSyn HILIC microparticles (Resyn Biosciences, Pretoria, SA), according to the manufacturer’s protocol. The HILIC beads selectively bind to proteins, facilitating the removal of interfering substances that may hinder trypsin activity as well as affect downstream MS analysis. A 96 deep-well plate was set-up by adding reagents and samples in rows A to G. The automated system has a 12-pin robotic magnet head with a disposable plastic comb, initially placed in Row H, that allows for the mixing and the transferring of reagents and samples between wells during the different steps carried out by the magnetic handling station. MagReSyn HILIC beads were washed in Row G and transferred to Row F for equilibration (1 min) followed by protein binding in Row E (30 min). Two successive washes in Rows D and C (1 min) were carried out to remove contaminants. Proteins were then digested through the addition of sequence grade modified trypsin (Promega, Madison, USA) at a 1:10 ratio, and the reaction was allowed to proceed for 2 h at 47 °C in Row A which was temperature controlled using a Kingfisher™ Duo Peltier heating block. After protein digestion with trypsin, the HILIC beads were removed from protein digests and transferred to the original storage position in Row G. Peptide solutions were harvested from Row A and transferred to 0.5 ml Eppendorf Protein LoBind tubes and vacuum dried at ambient temperature using a CentriVap (Labconco, Missouri, USA)^22^.

#### High pH reverse phase (HpHRP) fractionation

The Pierce Quantitative Colorimetric Peptide Assay (Thermo Fisher Scientific, Massachusetts, USA) was used to determine peptide concentrations post on-bead digestion. 20 µg of tryptic digest pooled from biological samples, were analysed using HpHRP. This was performed using a Dionex UltiMate 3000 RSLC (Thermo Fisher Scientific, Massachusetts, USA) coupled with an Acclaim PA II column (1.0 mm × 15 cm, C18, 3 µm, 120 Å) (Thermo Fisher Scientific, Massachusetts, USA). Mobile phase A contained 20 mM ammonium hydroxide in water (pH 9.6) and Mobile phase B contained 20 mM ammonium hydroxide (pH 9.6) supplemented with 80% acetonitrile in water. Peptide separation was done at 50 µl/min using a 35-min gradient (4–40% B). 30 fractions were collected every 30 secs from 12.5 to 27.5 min and then pooled to generate 10 concatenated fractions as per the following pooling scheme: (F1 = [1, 11, 21]; F2 = [2, 12, 22]; F3 = [3, 13, 23]; F4 = [4, 14, 24]; F5 = [5, 15, 25]; F6 = [6, 16, 26]; F7 = [7, 17, 27]; F8 = [8, 18, 28]; F9 = [9, 19, 29], F10 = [10, 20, 30]). Pooled fractions were vacuum dried using a CentriVap (Labconco, Missouri, USA) and stored at − 80 °C until further analysis.

#### LCMS-MS analysis

Pooled peptide fractions and individual plasma samples were re-suspended in 25 µl 2% acetonitrile/0.2% formic acid and spiked with iRT peptide standards (Biognosys, Zurich, Switzerland) as per manufacturer instructions. Analysis was performed on a Dionex Ultimate 3000 RSLC system (Thermo Fisher Scientific, Massachusetts, USA) coupled to a SCIEX 6600 TripleTOF mass spectrometer. Injected peptides (500 ng) were de-salted online using an Acclaim PepMap C18 trap column (75 μm × 2 cm) for 5.5 min at 5 μl/min using 2% acetonitrile/0.2% formic acid. Trapped peptides were separated on a Waters NanoEase™ C18 column (75 μm × 25 cm, 1.7 µm particle size). Peptides were eluted using a flow-rate of 300 nl/min and a gradient: 2–35% B over 45 min (A: 0.1% formic acid; B: 80% acetonitrile/0.1% formic acid). In DDA mode (pooled peptide fractions), precursor (MS) scans were acquired from *m/z* 400–1500 (2 + to 5 + charge states) using an accumulation time of 250 ms followed by 80 fragment ion (MS/MS) scans, acquired from *m/z* 100–1800 with 25 ms accumulation time each. For SWATH mode (individual plasma samples), precursor scan covered the range *m/z* 400 to 900 followed by 60 variable-width windows that overlapped by 0.5 Da, with fragment ions acquired from *m/z* 100–1800 with 25 ms accumulation time per window. See Supplementary Table 1 for summary of methods.

#### Data processing

##### Spectral library generation

DDA runs from pooled plasma were initially processed using Protein Pilot (v 5.0.1)^23^, where raw data (*.wiff) files were searched against the human UNIPROT database (20190609) of human protein sequences concatenated with a list of common contaminating proteins as well as the sequences of the Biognosys iRT peptide retention time standards. In the search settings, trypsin was selected as the proteolytic enzyme, iodoacetamide based alkylation and thorough search effort with biological modifications allowed, was also selected. A false discovery rate (FDR) analysis was set at 1% local FDR cut-off applied at PSM, peptide and protein levels. These were imported into Spectronaut™ 11 (Biognosys) to generate a spectral library using default settings. Briefly, the raw files (.wiff) were initially converted into Spectronaut™ (.HTRMS) file format to enable faster processing. Retention time prediction was set to dynamic iRT and correction factor for window was set at 1. Local mass calibration, MS1 and MS2 level interference correction was enabled. Cross run global normalisation, based on the algorithm by Callister and colleagues^24^, was enabled.

##### SWATH data extraction

Raw SWATH™ data files (.wiff) were imported into Spectronaut™ 11 (Biognosys Inc,) and extracted using the in-house generated spectral library. Briefly, dynamic iRT retention time prediction was used with the correction factor for window set at 1. The decoy method was set as scrambled database and the FDR was set at 0.01 (1%) at the peptide level. Spectronaut performed an unpaired comparison (two-sample t-test) between samples labelled according to experimental conditions, based on the log2 ratios of individual intensities of peptides of a protein. Kernel Density Estimator was selected as the P value estimator, corrected for multiple testing using the q-value threshold set at ≤ 0.01 to control the overall FDR^25^. A log2 fold change cut-off of 0.58 was used to select peptides for protein group quantitation. Cross run global normalisation was enabled, and individual runs were normalised using the median quantities of all peptides selected for normalisation.

##### Functional data annotation

A list of candidate proteins with associated average log_2_ ratios representing differential abundance (0.01 Q-value cut-off) was exported directly into Cytoscape v3.8.0 to generate a String enrichment analysis using the StringApp v1.5.1^26^. A protein query was generated using imported enrichment data from the String database using a confidence cut-off score of 0.7 for the interactions retrieved. This generated an enrichment table, including enriched terms with corresponding gene components.

## Results

### Hormone therapy treated T47D cells show greater propensity to upregulate EMT markers on exposure to whole blood

In this study Anastrozole or Tamoxifen-treated breast cancer cells were exposed to whole blood for 2.5 min to facilitate the interaction between cancer cells and whole blood components. We have previously identified this duration of exposure as sufficient to induce platelet activation and elicit changes to cancer cell gene expression^13,19^. In the present study, following exposure to whole blood (WB), T47D cells presented with consistently higher relative gene expression levels of *ACTB3*, *TGFβ1*, *CDH1*, *Vimentin*, *AKT1* and *BECN1* and higher absolute levels of *ACTB3*, *ESR1* and *ESR2* in comparison to MCF7 cells (Tables 2, 3).

**Table 2:**
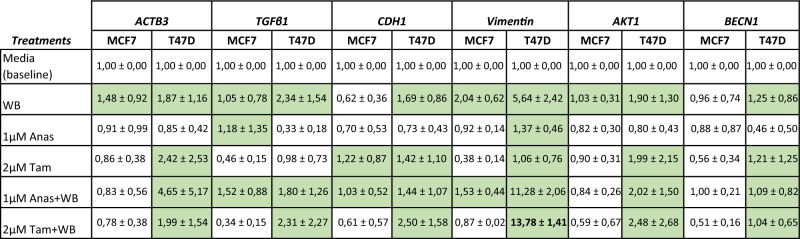
Fold change in gene expression (2^ΔΔCt^) of selected genes; *ACTB3*, *TGFβ1*, *CDH1*, *Vimentin*, *AKT1* & *BECN1* which are involved in various tumour processes.

The media control was used as a baseline control and fold change was determined in relation to the media control with values above 1 indicating upregulation and values below 1 indicating down regulation of genes. T47D cells generally presented with higher expression of the indicated genes following exposure to most treatments (except 1 µM anastrozole) compared to MCF7 cells. *Vimentin* expression in T47D cells treated with 2 µM tamoxifen and exposed to whole blood (WB) increased significantly compared to T47D cells with WB exposure alone and those treated with 1 µM Anastrozole.

Green background = upregulated genes in relation to baseline, white background = downregulated genes in relation to baseline. Bold = significantly upregulated (*p* < 0.05) in relation to whole blood only and 1 µm Anas + WB treatment in T47D cells. Data presented as (mean ± SD).

**Table 3:**
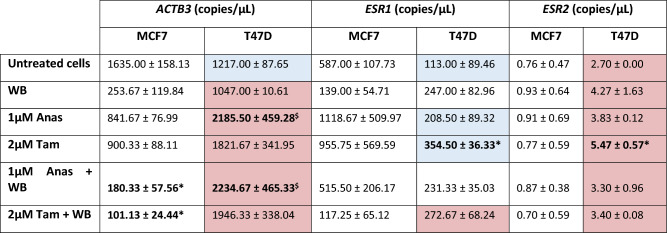
Absolute concentrations of ACTB3, ESR1 and ESR2.

Hormone-therapy combined with exposure to whole blood significantly reduced *ACTB3* in MCF7 cells whereas T47D cells upregulated *ACTB3*, particularly under Anastrozole treatment. Tamoxifen treatment induced significantly higher *ESR1* and *ESR2* expression in T47D cells compared to untreated cells. Nevertheless, *ESR1* expression was more stable in T47Ds across the different treatment groups, with *ESR2* expression more stable in MCF7 cells. With the exception of *ACTB* in untreated T47D cells, matched analysis showed T47 cells significantly upregulated *ACTB3* and *ESR2* compared to MCF7 cells in all treatment groups*,* only significantly raising *ESR1* expression in response to Tamoxifen treatment and WB exposure.

Bold p < 0.05* compared to untreated cells; $ compared to WB treatment. For T47D cells: highlighted blue p < 0.05, downregulated compared to matched treatment with MCF7; highlighted pink, p < 0.05 upregulated compared to matched treatment with MCF7. Data presented as (mean ± SD).

Exposure to WB following treatment with Anastrozole and Tamoxifen, increased *Vimentin* expression significantly (p < 0.05) (Table 2) in T47D cells. Of the genes associated with EMT, no marked alterations were identified relating to the expression of *CDH1 *(*p* > 0.05), but qPCR analysis showed *ACTB3* was marginally raised under hormone-therapy in T47D cells (p > 0.05). We further assessed this change with ddPCR where absolute quantification established that not only did Anastrozole treatment increase *ACTB3* levels in T47D cells; but that these levels were significantly raised following exposure to whole blood compared to MCF7 cells (Table 3). MCF7 cells downregulated *ACTB3* (p < 0.05) expression, when treated with Tamoxifen and Anastrozole and exposed to whole blood, compared to untreated cells (Table 3). Genes associated with autophagy, *AKT* and *BECN1* showed stable expression in both cell lines. Notably, exposure to whole blood following treatment with Anastrozole raised *TGFβ1* expression in the MCF7 cell line, while the same was observed for T47D cells under Anastrozole and Tamoxifen treatment, albeit non-significant (Table 2).

Tamoxifen’s effects on T47D cells were notable causing upregulation (p < 0.05) of *ESR1* and *ESR2* compared to untreated cells (Table 3). The phenotype of T47D cells differs to that of MCF7 cells in that the former presents with greater *ESR2* expression, and the latter, greater *ESR1* expression (Table 3). Matched analysis yielded interesting results, in that all treatments significantly upregulated *ACTB3* and *ESR2* in T47D cells compared to MCF7 cells. T47D cells only significantly raised *ESR1* expression in response to Tamoxifen treatment and whole blood exposure; with notable downregulation of the gene induced by Anastrozole and Tamoxifen treatment alone, compared to MCF7 cells.

**Figure 1:**
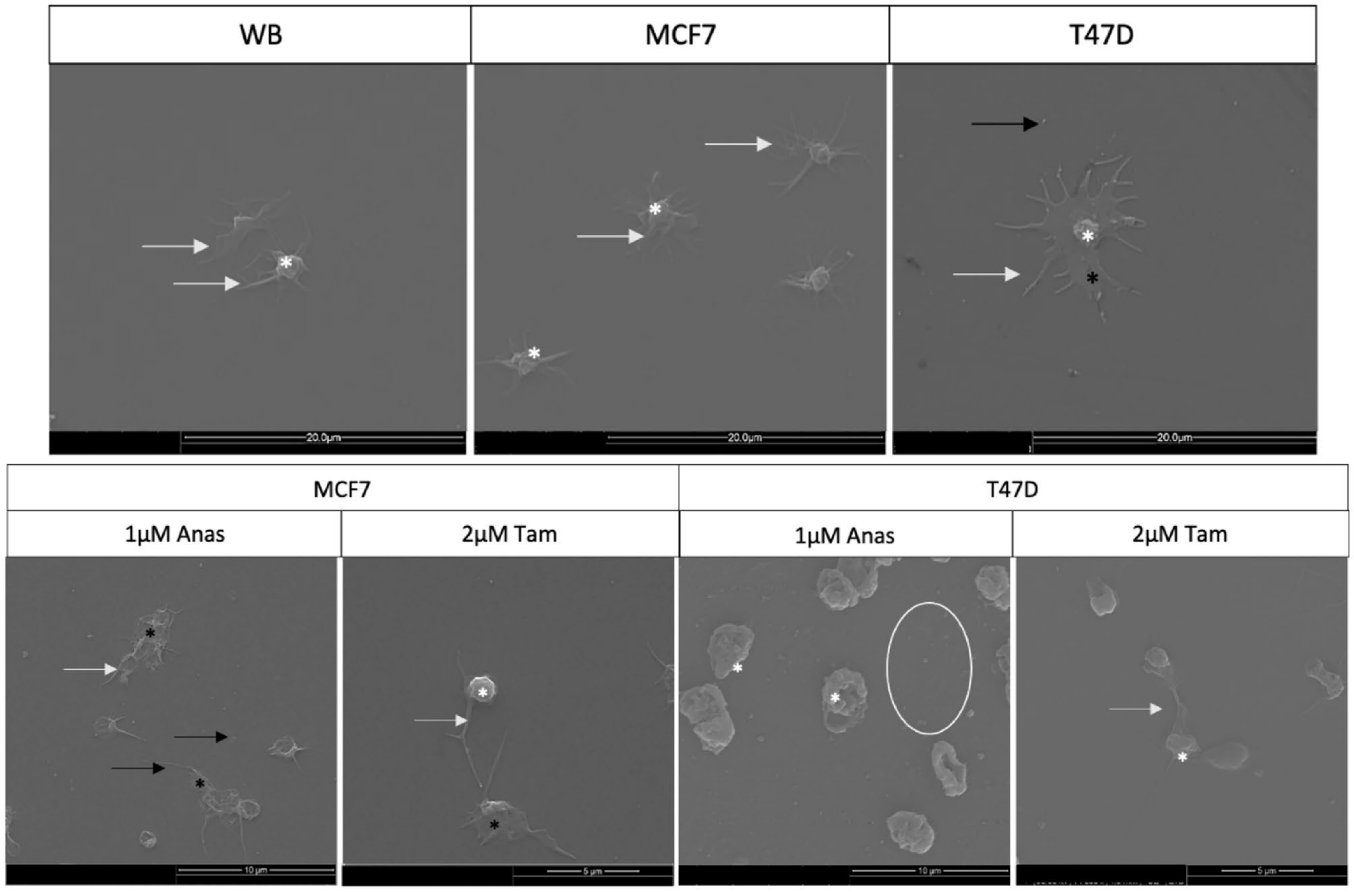
Platelet activation in WB exposed to hormone therapy-treated breast cancer cell lines. Untreated MCF7 and T47D cells induced platelet activation in WB. Note pseudopodia extension and hyalomere spread, more so than that evidenced in control WB. Exposure of WB to either Anastrozole or Tamoxifen-treated breast cancer cells resulted in further platelet activation, aggregation and membrane disruption. Key: black *membrane folds, white *hyalomere spread, white arrowextending filipodia, black arrowmicroparticles.

Corresponding changes in platelet ultrastructure following exposure to hormone therapy-treated breast cancer cells, confirmed that hormone-therapy enhanced the capacity of MCF7 and T47D cells to induce platelet activation (Fig. 1). Extending pseudopodia indicated early stages of platelet activation, with spread hyalomere indicative of later stages. Exposure to hormone-therapy treated cells resulted in more platelet aggregation in both cell lines. However, T47D cells induced greater disturbances in the platelet membrane. A fibrin film and microparticles were also evidence of hypercoagulation.

### Hormone therapy-induced changes in secreted protein profiles from breast cancer cells

**Figure 2:**
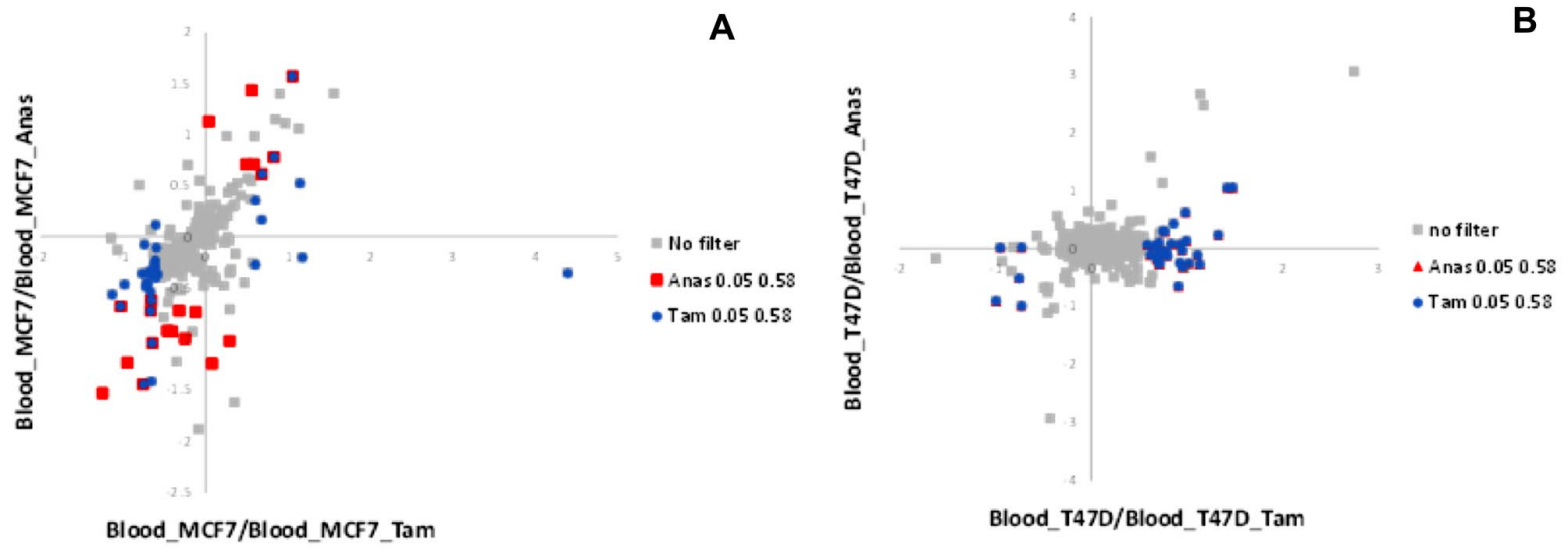
Scatter plot indicating dysregulated proteins in plasma exposed to MCF7 (**A**) and T47D (**B**) that were treated with Tamoxifen and Anastrozole. Grey dots indicate the background of all identified proteins for both treatments. Blue indicates dysregulated proteins after treatment with Tamoxifen and red dots indicate dysregulated proteins after treatment with Anastrozole at a minimum fold rate of 1.5 ≥ and maximum adjusted Q-value of ≤ 0.05. MCF7 cells showed a greater propensity to alter the secreted protein profile under both treatment modalities in comparison with T47D cells.

**Figure 3:**
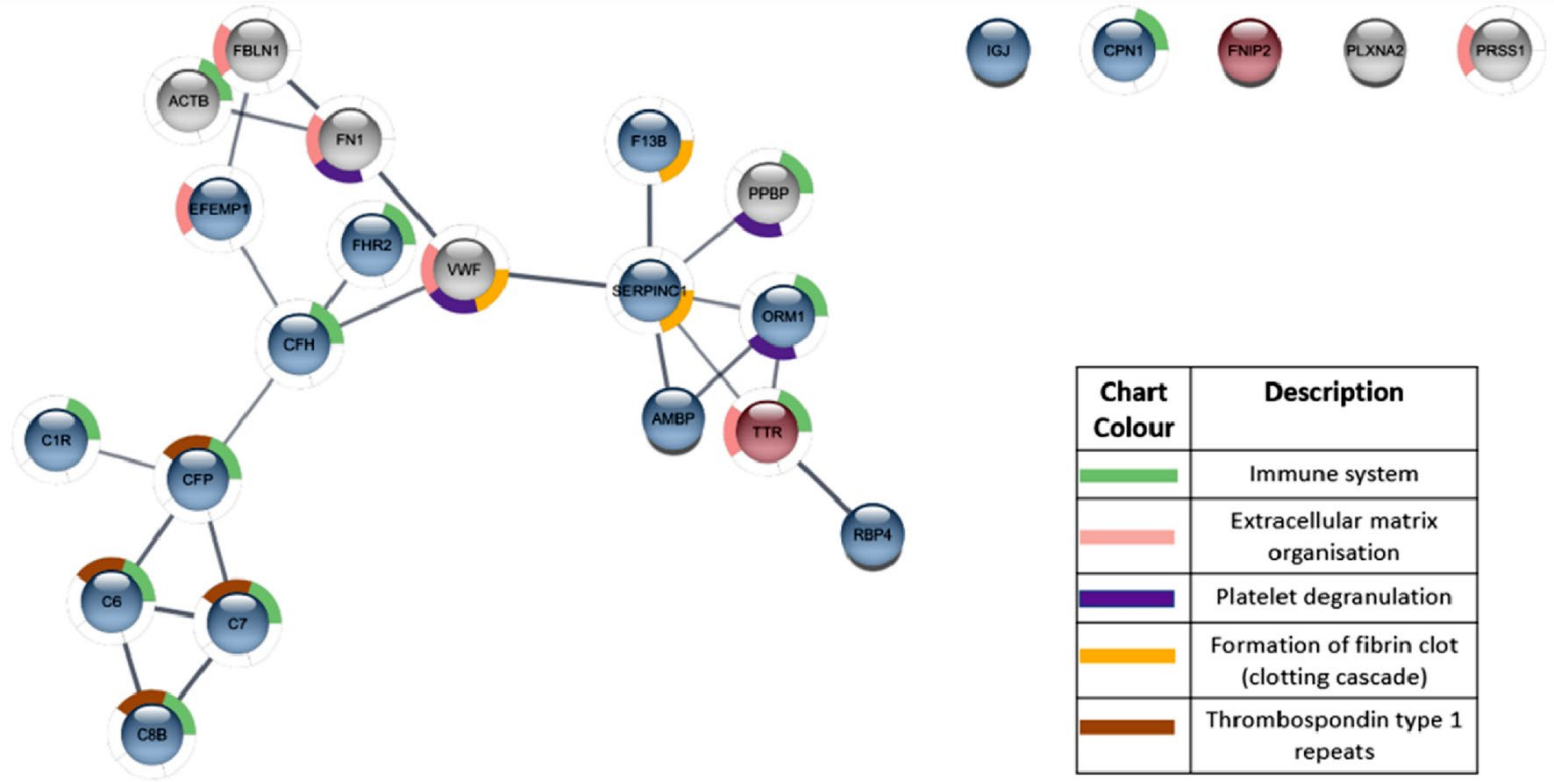
String plot indicating enriched pathways and the relationship between the genes that were dysregulated after treatment with Tamoxifen in cell line MCF7. Red indicates upregulation, blue indicates downregulation and grey indicates no significant difference after drug treatment. Minimum fold change of 1.5 ≥ and maximum adjusted Q-value of ≤ 0.05.

Plasma samples obtained from WB exposed to hormone-therapy treated breast cancer cells were analysed for short term changes in the protein profiles either secreted by the cancer cells or released by platelets during the activation process. Using SWATH-MS we were able to quantify 257 protein groups and 2833 peptides. Fold change, calculated using a linear mixed-effects model, permitted the determination of relative abundance of each quantifiable protein between plasma samples obtained from whole blood exposed to Anastrozole or Tamoxifen-treated breast cancer cells, and the corresponding control, plasma exposed to untreated breast cancer cells. A minimum fold change ≥ 1.5 and maximum, multiple hypothesis adjusted, Q-value ≤ 0.05 was used to filter proteins that were significantly different between the experimental and control samples. MCF7 cells treated with either hormone-therapy (Tamoxifen or anastrozole) were prone to modulate a greater number of proteins than T47Ds (Figs. 2, 3, Supplementary Fig. 1, Supplementary Table 2). However, both cell lines were more affected by Tamoxifen treatment.

**Figure 4:**
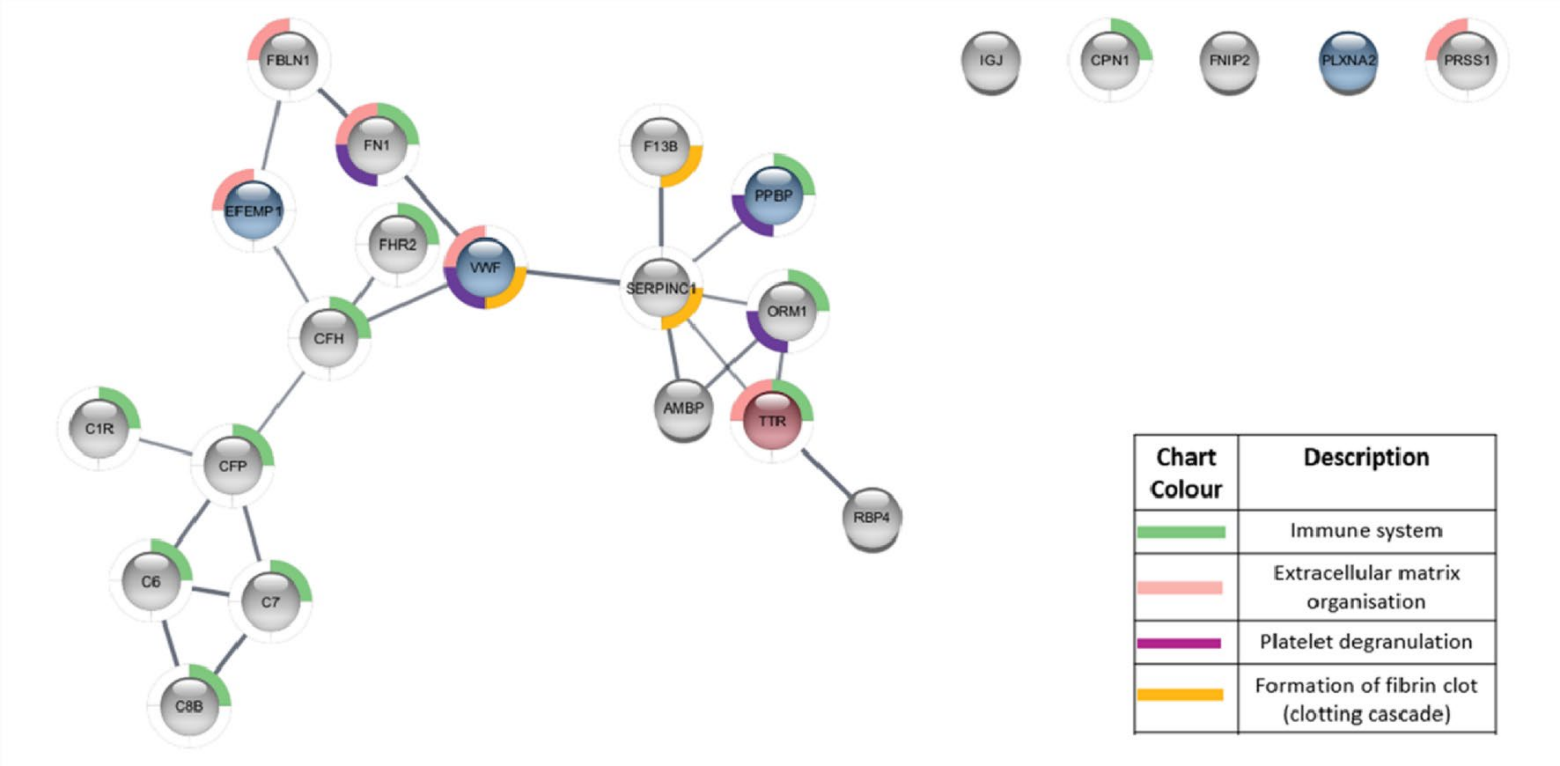
String plot indicating enriched pathways and the relationship between the genes that were dysregulated after treatment with Anastrozole in cell line MCF7. Red indicates upregulation, blue indicates downregulation and grey indicates no significant difference after drug treatment. Minimum fold rate of 1.5 ≥ and maximum adjusted Q-value of ≤ 0.05.

Specifically, dysregulated proteins detected were primarily secreted proteins, with MCF7 cells showing a greater propensity to alter the secreted protein profile following both Anastrozole or Tamoxifen treatment than T47D cells (Fig. 2). Hormone-therapy of MCF-7 cells followed by whole blood exposure, resulted in dysregulation of proteins associated with immune activation and extracellular matrix organisation pathways (Figs. 3, 4). The downregulation of thrombospondins (Fig. 3) was of particular concern under Tamoxifen treatment (Fig. 3), and not identified under Anastrozole treatment. Under both therapies, engagement of the clotting cascade and platelet degranulation (Figs. 3, 4) echoed morphological analyses.

When assessing MCF7 cells specifically, the reduction of detected vWF (Anastrozole-treated MCF7 samples) and AT3 (Tamoxifen-treated MCF7 cell samples), was of particular interest (Figs. 2, 3, Supplementary Table 2). Data reported by the Human Protein Atlas database (www.proteinatlas.org) indicates that breast tumour stroma shows positivity for vWF with mean plasma concentrations of AT3 relatively high. These results point to an induction of a hypercoagulable environment, supported by the morphological results. It is suggested that released vWF may have bound to platelets, with lower levels of AT3 permitting thrombin activity, enhancing platelet activation and aggregation (Fig. 3, Supplementary Table 2). Similarly, downregulation of FN1, FBLN1 (also in Tamoxifen-treated T47D/MCF7 samples and Anastrozole-treated MCF7 samples) and EFEMP1 (Tamoxifen and Anastrozole-treated MCF7 samples) may indicate their integration into platelet clot stabilisation and fibrin formation (Fig. 4, Supplementary Table 2). In Tamoxifen-treated MCF7 samples, the downregulation of the JCHAIN and C1RL, which are reported by the Human Protein Atlas database to be associated with favourable outcomes in breast cancer, is of concern; however, IGKV-4, which is favourable in breast cancer, was upregulated (Figs. 3, 4, Supplementary Table 2).

**Figure 5:**
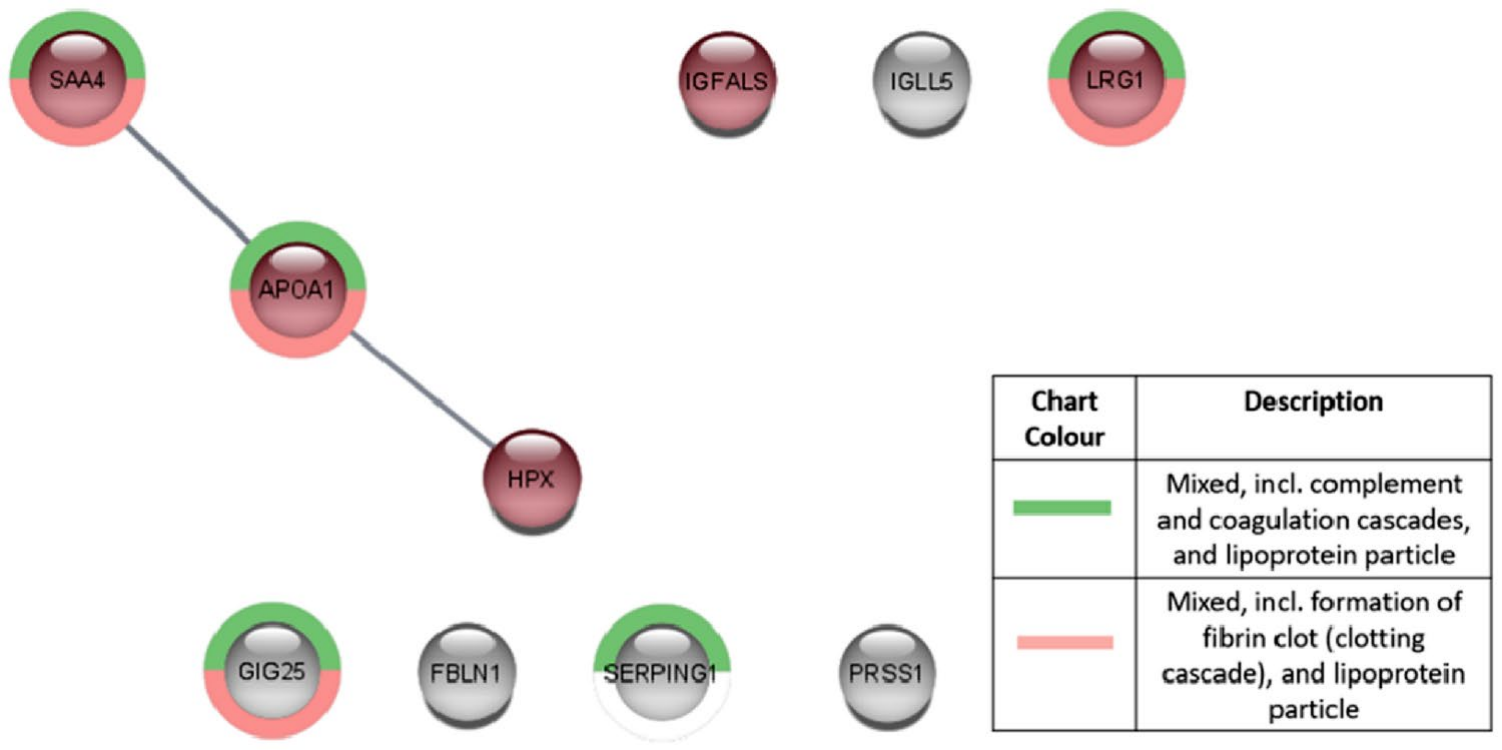
String plot indicating enriched pathways and the relationship between the genes that were dysregulated after treatment with Tamoxifen in cell line T47D. Red indicates upregulation, blue indicates downregulation and grey indicates no significant difference after drug treatment. Minimum fold rate of 1.5 ≥ and maximum adjusted Q-value of ≤ 0.05.

In T47D cells, Tamoxifen treatment enhanced pathways associated with complement factors and the coagulation cascade (Fig. 5), but no pathways were enriched for Anastrozole treatment. Specifically, Tamoxifen upregulated SERPINA3 and IGLV7-43, while Anastrozole-treated T47Ds downregulated IGCL3 (Supplementary Table 2) which is indicated by the Human Protein Atlas as being associated with favourable outcome in breast cancer. APOA1 was also upregulated under Tamoxifen treatment, but no prognostic value in breast cancer has been found yet.

**Figure 6:**
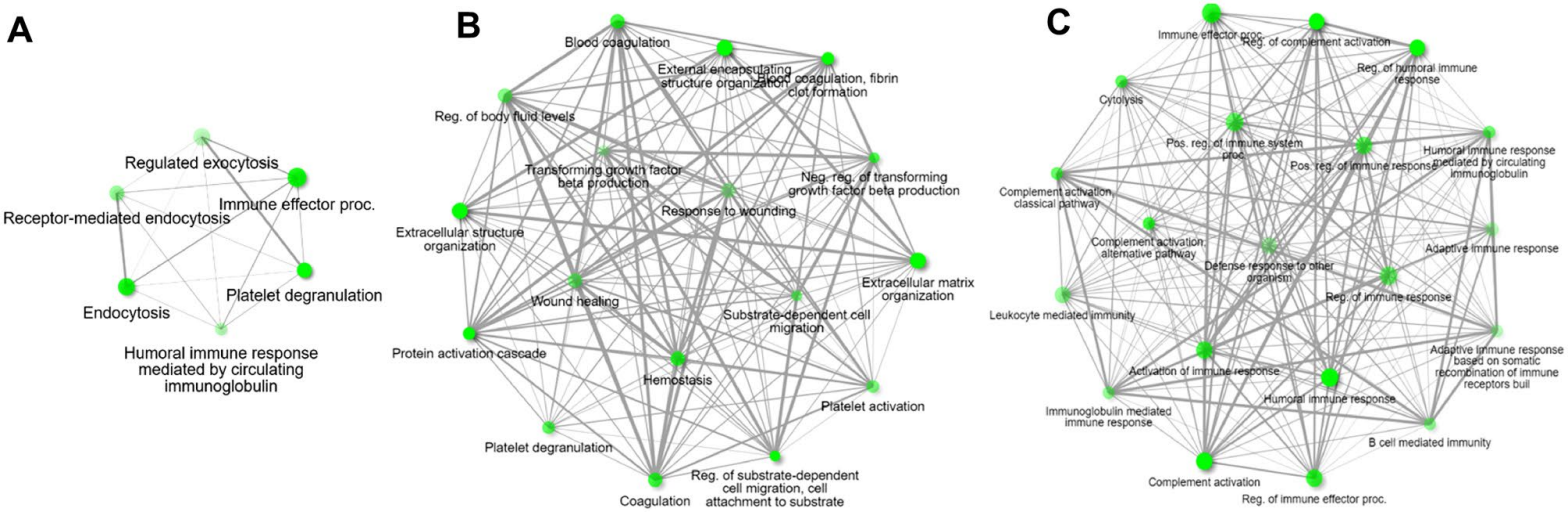
Interaction network indicating enriched pathways and the relationship between the biological processes for proteins that were dysregulated in plasma after (**A**) exposure to Tamoxifen-treated T47D; (**B**) exposure to Tamoxifen-treated MCF7; (**C**) exposure to Anastrozole-treated MCF7 cells. No pathways could be enriched for the effects induced by Anastrozole-treated T47D cells. Minimum fold rate of 1.5 ≥ and maximum adjusted Q-value of ≤ 0.05. ShinyGO was used to generate the plot. The software connects Two pathways (nodes) if they share 20% (default) or more genes. Darker nodes are more significantly enriched gene sets. Bigger nodes represent larger gene sets. Thicker edges represent more overlapped genes.

Even with short exposure times, differentially expressed proteins highlighted several biological processes illustrated by interaction networks. The top pathways enriched under Tamoxifen treatment included for both cell lines; platelet degranulation processes (Fig. 6) that would be subject to platelet activation (Fig. 1), and engagement of the immune system, particularly immunoglobulin release, the humoral arm and complement pathway. Notable enriched pathways induced by MCF7 cells under both hormone-therapies, included complement activation, with Tamoxifen treatment specifically associated with upregulation of pathways linked to cell migration, wound healing and coagulation. Top downregulated pathways involved in negative regulation and production of TGFβ were also evident in Tamoxifen-treated MCF7 samples (Fig. 6B). This must be considered in light of TGFβ sources from both the tumour cells (Table 2) and platelets. An interaction network for the effects of Anastrozole could only be generated for MCF7 cells, which showed greater responses to this drug than T47D cells. Top upregulated pathways included those associated with innate immunity, complement activation, as well as humoral immunity.

## Discussion

Crosstalk between platelets, platelet-derived microparticles and tumour cells has been identified within the tumour bed, with pro-tumorigenic actions dominating the literature^2–6^. During escape from the primary site, tumour cells are postulated to undergo epithelial-mesenchymal transition, becoming more invasive and migratory as they prepare to enter the bloodstream. Modulation or evasion of immunity is necessary for successful dissemination and establishment of tumours at secondary sites. Studies have recapitulated the impact of platelets in multiple pro-tumorigenic processes including driving EMT in tumour cells^1,7,8^, preparation of the pre-metastatic niche^9^, tumour cell-transendothelial migration^10^, and protection of tumour cells within the vasculature^11^. To understand this phenomenon, we have used a simple co-culture system to investigate the crosstalk between platelets in whole blood and breast cancer cell lines. We have previously shown that a short duration of exposure (2.5 min) was sufficient for breast cancer cell lines to induce platelet activation, as identified ultrastructurally and using flow cytometry^8,13,18,19^. This short duration of exposure is predicated on the rapidity of platelet activation. Studies indicate that under agonist stimulation, release of stored Ca^2+^, necessary for the activation process, occurs well within 60 seconds^27^. Expression of CD41 (integrin α_IIb_β3), a marker of early platelet activation, is reported to occur within 2 min depending on the agonist used^28^, and reflects engagement of the degranulation process within that short time-period^29^. In this study we delved further into the relationship by assessing the effects of whole blood exposure on the capacity of hormone-therapy treated breast cancer cells to modulate EMT and pro-survival tumour characteristics, and their capacity to alter the secreted protein profile for either pro- or anti-tumour processes. While both MCF7 and T47D cells are classified as hormone-dependent, luminal phenotype breast cancer, their response to therapy appears markedly different and appears underwritten by the variance in their phenotype^8,18,19^.

In this study we show that T47D cells present with lower *ESR1* expression and higher *ESR2* expression (albeit low levels) than MCF7 cells. ERα (or pan-ER) is typically used as a clinical diagnostic parameter, with hormone-therapy targeted at reducing pro-tumorigenic oestrogen-dependent signalling^29^. As such, the efficacy of hormone-therapy is predicated on the presentation of hormone-receptors. Specifically, Anastrozole limits the production of oestrogen via inhibiting the function of aromatase, whereas Tamoxifen prevents the binding of oestrogen to ER^18,29^. However, it must be noted that constitutive ER signalling can also be induced by oestrogen-independent methods^30^. Moreover, luminal phenotype breast tumour cells can downregulate ER presentation as an evasive strategy, during EMT, or as part of acquiring a resistant phenotype^31,32^. ERα and ERβ are approximately 60% homologous with respect to DNA binding and ligand binding sites; however, they have been postulated to have different functionality^33^. In breast cancer, ERα activation has a well-known association with enhancing proliferation and tumorigenesis. ERβ is postulated to prevent ERα action, exerting anti-proliferative effects; with high expression of ERβ typically associated with better patient outcomes^34^. However, some studies have found contradictory results, which may be linked to the expression of isoforms of ERβ; for example, cytoplasmic expression of ERβ2 is linked to poor response to chemotherapy and poor overall survival^33^. We had previously shown that Tamoxifen and Anastrozole treatment, particularly in T47D cells, largely increased both ERα and ERβ expression in both nuclear and cytoplasmic compartments, enhanced by exposure to whole blood^8,19^. In the present study, T47D cells increased the levels of *ESR2* response to hormone-therapy, particularly Tamoxifen, which may reflect the heightened sensitivity to Tamoxifen conferred by innate ERβ overexpression^33^. ERβ overexpression has also been linked with an increase in autophagy^33^; however, in the present study there was no concomitant increase in *BECN1* that would indicate enhanced autophagy.

The overexpression of ERβ has also been linked in breast cancer cells to a reduction in Akt signalling and thus a reduction in pro-tumorigenic processes^35^; however, in the present study T47D cells consistently presented higher *AKT* expression that was enhanced by whole blood exposure. This may point to a more aggressive, or more mesenchymal tumour profile^32^. Additionally, downregulation of ERα has been linked to the induction of a more mesenchymal phenotype in MCF7 cells^36^. In the present study while whole blood exposure to hormone-therapy treated breast cancer cells decreased *ESR1* expression substantively, corresponding changes in EMT-associated genes indicated higher levels of *CDH1* and *vimentin* in T47D cells than MCF7 cells. In addition, reduced levels of *CDH1* and *vimentin* expression were observed in Tamoxifen-treated MCF7 cells exposed to whole blood. EMT is typically associated with the downregulation of *CDH1*^37^, in our culture system this reduction was observed in MCF7 cells more than in T47D cells; however, both hormone-therapies and whole blood exposure significantly increased *vimentin* and *TGF-β1* expression in T47D cells, indicative of partial-EMT^35,38^. Previously we demonstrated that *TGF-β1* and *vimentin* genes were upregulated in MCF7 cells exposed to whole blood and platelet-rich plasma for a longer duration of 5 minutes^13^. In the present study, under a shorter period of exposure (2.5 min), induction of EMT processes were also highlighted by MCF7 cells treated with both hormone-therapies and exposed to whole blood. Interaction networks showed enrichment of pathways involved in wound healing, TGF-β production and extracellular matrix reconfiguration, dysregulation of which are associated with tumour progression^10,13,30,32,36,39^. Additionally, *ACTB3*, a well-known and often used reference gene was shown by the digital droplet PCR technique which allows absolute quantification, to also be upregulated. A pan-cancer study of *ACTB* of patient cancer data has illustrated that its upregulation is associated with EMT^40^. The results of the present study suggest that hormone-therapies and whole blood exposure induced pro-survival processes and a more aggressive phenotype, particularly in T47D cells. This was also highlighted by the induction of platelet activation, where treated T47D cells were more prone to cause platelet membrane damage. But similarly, Anastrozole and Tamoxifen treatment enhanced the capacity of MCF7 cells to induce features of hypercoagulation, including thrombin secretion and fibrin film formation^8,18^.

The reciprocal interactions between breast cancer cells and platelets are mediated, to a large degree, by secreted factors produced by both cell types. We assessed this by determining the secreted protein profile in plasma following exposure of whole blood to hormone-therapy treated breast cancer cells. Notably, MCF7 cells showed a greater propensity to alter the secreted protein profile, in comparison to T47D cells which conversely had shown greater adaptation in terms of gene expression, to cell survival and pro-tumorigenic processes. Moreover, the variance in induced platelet activation between both cell lines must also be noted. It is thus expected that the duration of co-culture for T47D cells may need to also be increased to permit translation and detection of secreted proteins. Important to consider is that the whole blood used in this study was not depleted of white blood cells. As such we must consider the duration required in which cells of the immune system may be activated in our culture system. A recent study employing optically trapped macrophages demonstrate reactive oxygen species production on activation in 3 mins^41^; neutrophil activation with selected stimuli in 1–2 h^42^, and priming for T cells ranging from 2 to 20 h ^43^. The duration of exposure of whole blood to tumour cells in this study was only 2.5 min and thus while we postulate it unlikely that white blood cells were activated, we cannot definitively exclude such influences on tumour cell behaviour or platelet activation. Further investigations are thus warranted.

Tumour cells can induce platelet activation via the secretion of major factors involved in the coagulation cascade including thrombin, von Willebrand factor, tissue factor and ADP^3^. Induced platelet aggregation is a major concern in inducing thrombosis in cancer patients, with degranulation of platelets also implicated in the release of pro-tumorigenic molecules^44^. In the present study downregulation of secreted von Willebrand factor and anti-thrombin was postulated to be associated with a positive feedback loop that illustrated platelet activation, particularly by MCF7 cells under both hormone-therapies. Morphologically, platelets showed clear extension of pseudopodia and hyalomere spread, morphological changes indicative of early degranulation^45^. We also illustrated through proteomic analysis, pathways that were enriched following both Tamoxifen and Anastrozole treatment of MCF7 cells, included those involved in platelet degranulation and initiation of the clotting cascade.

Tumour cells were able to modulate fibronectin-1, fibulin-1 and EGF-containing fibulin-like extracellular matrix protein 1, indicating their integration into platelet aggregates and fibrin formation (Human Protein Atlas, www.proteinatlas.org). Moreover, these proteins are also associated with the induction of EMT and induction of angiogenesis^39,46^. This was combined with the enrichment of pathways associated with downregulation of proteins involved in extracellular matrix reorganisation, including thrombospondins (TSP), under Tamoxifen treatment. Thrombospondins, a family of extracellular matrix proteins have considerable implications in tumour progression, and depending on microenvironmental cues may stimulate or inhibit tumour progression^47^. TSP-1 is a constituent of platelet α-granules, and even low concentrations have been associated with enhancing breast cancer proliferation and invasion^47,48^. Moreover, in concert with TGF-β, regulation of which in this study was illustrated as a major player in MCF-7 response to Tamoxifen, a TSP-1 axis has been implicated in modulating establishment of the pre-metastatic niche^49^.

In contrast, T47D cells, while inducing later stages of platelet activation did also damage cellular membranes to an extent and caused fibrin films. This capacity for inducing a hypercoagulatory environment was further highlighted by string analysis showing upregulation of proteins involved in fibrin clot development and the coagulation cascade. This echoes previous results in our lab, indicating that T47D response to particularly Tamoxifen resulted in upregulation of the late stage platelet activation marker, CD63^8^. Additionally, our results explain the mechanism by which Tamoxifen induces thrombosis in cancer patients, a well-known risk of this drug^17^, as well as processes by which tumour cells may adapt, acquiring more malignant phenotypes under the selective pressures of drug treatments^19^. Processes that were also reflected by proteomic analysis included enrichment of pathways associated with humoral immunity, for both cell lines but particularly under Tamoxifen treatment. T47D cells particularly upregulated a number of immunoglobulins. While Ig expression is traditionally associated with humoral immunity, these evolutionary ancient molecules have been shown to be expressed on several tumour cells derived from epithelial cancers^12^. Tumour-derived IgG has been associated with promoting aggressive tumour behaviour including, migration and invasion; immune escape; and the induction of platelet activation and aggregation although the mechanisms remain elusive^12,49^. Moreover, Ig-bound molecules including thyroid hormone-binding protein, upregulated following hormone-therapy of MCF7 cells, and apolipoprotein-1, upregulated following Tamoxifen treatment of T47D cells, are suggested to hold predictive value in pancreatic adenocarcinoma response to therapy^50^, although their utility in breast cancer remains unknown^51^. Additionally, in the present study, enrichment of the complement pathway was detected, primarily in plasma exposed to Anastrozole-treated MCF7 cells. While Ig may induce complement activation, the exact mechanism of induction and the role of complement in the tumour microenvironment has yet to be fully elucidated^51,52^. Notably, tumour cells themselves are capable of secreting complement proteins, postulated to assist in creating an immunosuppressive environment that would facilitate immune evasion and metastasis^51,52^. In the present study, no pathways could be enriched for the plasma samples that had been exposed to Anastrozole-treated T47D cells; however, individual proteins that were dysregulated in this group include a substantive decrease in IGLC3, with low expression typically regarded as a favourable prognostic marker in breast cancer. However, we suggest that T47D cell response to therapy and whole blood, may require a greater duration of co-culture to elicit a detectable secreted response, given our understanding of their prolonged stress response in the face of survival pressures^53^.

## Conclusions

This co-culture method has highlighted the adaptability of tumour cells to treatment, and to engagement with whole blood to result in a pro-tumorigenic response and a hypercoagulatory state. We have illustrated that the secretome of breast cancer cells can be altered by hormone-therapy, but that this may be subject to the subphenotype of the cell line, notwithstanding the impact of platelet activation. Further research and the development of more sophisticated co-culture systems are required to recapitulate the reciprocal interactions between platelets and tumour cells to better understand tumorigenesis. In addition, deeper plasma profiling, using abundant protein depleted and/or vesicle enriched strategies, will likely reveal additional secretory proteins related to tumour cells–platelet interactions.

### Supplementary Information

Supplementary Information.

## Data Availability

The Authors will make datasets available on request subject to requirements from the Human Research Ethics Committee, University of the Witwatersrand. Please contact tanya.augustine@wits.ac.za.
