# Supplementary material for: Secretomics reveals hormone-therapy of breast cancer may induce survival by facilitating hypercoagulation and immunomodulation in vitro
Source: Sci Rep. 2024 Jan 17;14:1486. doi: 10.1038/s41598-023-49755-1 (PMC10794708; doi:10.1038/s41598-023-49755-1)
Supplement: Supplementary file 1 — Supplementary Information. [file 41598_2023_49755_MOESM1_ESM.docx]

**Supplementary Table 1**: Summary of Spectronaut™ experimental settings for quantitation.

| **Interference Correction** | Yes |
| --- | --- |
| Exclude All Multi-Channel Interferences | Yes |
| MS1 Min | 2 |
| MS2 Min | 3 |
| **Proteotypicity Filter** | None |
| **Major (Protein) Grouping** | By Protein Group Id |
| **Minor (Peptide) Grouping** | By Stripped sequence |
| **Major Group Quantity** | Mean peptide quantity |
| **Major Group Top N** | Yes |
| Max | 3 |
| Min | 1 |
| **Minor Group Quantity** | Mean precursor quantity |
| **Major Group Top N** | Yes |
| Max | 3 |
| Min | 1 |
| **Quantity MS-Level** | MS2 |
| **Quantity Type** | Area |
| **Data Filtering** | Q-value |
| **Cross Run Normalisation** | Yes |
| Normalisation Strategy | Global Normalisation |
| Normalise on | Median |
| Row selection | Automatic |


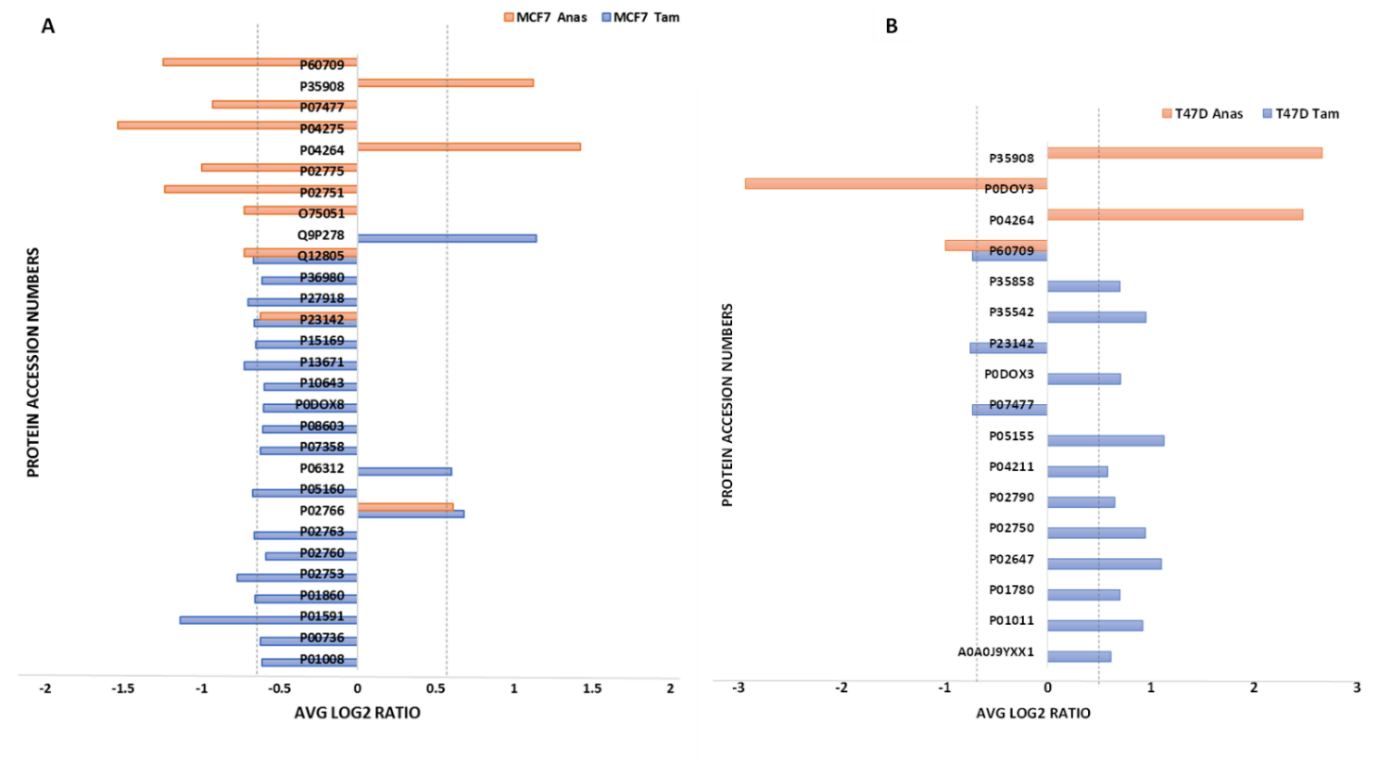
 **Supplementary Figure 1: Bar graph indicating up and down-regulated proteins in cell lines MCF7 (A) and T47D (B) after treatment with Tamoxifen (blue) and Anastrozole (orange).** Dotted line indicates minimum fold change of up-regulated proteins (+)/down-regulated proteins (-) ≥. 1.5. The maximum adjusted Q- value was set to ≤ 0.05.

**Supplementary Table 2:** Differentially expressed proteins by group with fold change. Function and association with cancer as described in the Human Protein Atlas presented.

| **Uniprot ID** | **Uniprot Name (gene)** | **Fold Change** | | **Function** | **Human Protein Atlas – cancer associations** | **Link** |
| --- | --- | --- | --- | --- | --- | --- |
|  |  | **MCF7-Tamoxifen** | **MCF7- Anastrozole** |  |  |  |
| ***P01008*** | Anti-thrombin (AT3/SERPINC1) | -0.614007447 |  | Inhibits thrombin, enhanced in the presence of heparin | Secreted. Mean plasma concentrations in breast cancer (pan-cancer cohort) higher than colorectal cancer but not prognostic | https://www.proteinatlas.org/ENSG00000117601-SERPINC1 |
| ***P00736*** | Complement C1r subcomponent (C1RL) | -0.623576409 |  | Classical pathway of the complement system | Secreted. Favourable prognostic marker in breast cancer | https://www.proteinatlas.org/ENSG00000139178-C1RL |
| ***P01591*** | Immunoglobulin J chain (JCHAIN) | -1.133522772 |  | Joins monomers of IgM or IgA | Secreted. Enhanced in breast cancer (favourable) | https://www.proteinatlas.org/ENSG00000132465-JCHAIN |
| ***P01860*** | Immunoglobulin heavy constant gamma 3 (IGHG3) | -0.658410368 |  | Constant region of immunoglobulin heavy chains | Low cancer specificity but a prognostic marker in renal cancer (unfavourable) and head and neck cancer (favourable) | https://www.proteinatlas.org/ENSG00000211897-IGHG3 |
| ***P02753*** | Retinol-binding protein 4 (RBP4) | -0.769169144 |  | Mediates retinol transport in blood plasma | Secreted. Mean plasma concentrations in breast cancer higher than colorectal cancer. Positivity in breast cancer but not prognostic. Poor prognosis in endometrial cancer. | https://www.proteinatlas.org/ENSG00000138207-RBP4 |
| ***P02760*** | Alpha-1-microglobulin (AMBP) | -0.588008286 |  | Antioxidant | Secreted. Upregulated in ovarian and endometrial cancer (favourable prognosis). Similar plasma concentrations in breast cancer (prognosis unknown) | https://www.proteinatlas.org/ENSG00000106927-AMBP |
| ***P02763*** | Alpha-1-acid glycoprotein 1 (ORM1) | -0.660240599 |  | Transport protein | Secreted. Unfavourable prognostic marker in renal cancer | https://www.proteinatlas.org/ENSG00000229314-ORM1 |
| ***P02766*** | Thyroid hormone-binding protein (TTR) | 0.68044777 | 0.611959397 | Transport protein | Secreted. Not prognostic in breast cancer but enhanced expression. | https://www.proteinatlas.org/ENSG00000118271-TTR |
| ***P05160*** | Coagulation factor XIII B chain (F13B) | -0.670146265 |  | Regulate the rate of transglutaminase formation by thrombin | Secreted. Favourable prognostic marker in liver cancer | https://www.proteinatlas.org/ENSG00000143278-F13B |
| ***P06312*** | Immunoglobulin kappa variable 4-1 (IGKV-4) | 0.60402818 |  | V-segment of variable domain involved in humoral immunity | Secreted. Favourable prognostic marker in breast cancer | https://www.proteinatlas.org/ENSG00000211598-IGKV4-1 |
| ***P07358*** | Complement component C8 beta chain (C8B) | -0.623172744 |  | Component of the membrane attack complex (MAC) - key role in the immune response by forming pores in target cell membranes | Secreted. Favourable prognostic marker in liver cancer. Not prognostic in breast cancer but tissue shows positivity | https://www.proteinatlas.org/ENSG00000021852-C8B |
| **Uniprot ID** | **Uniprot Name (gene)** | **Fold Change** | | **Function** | **Human Protein Atlas – cancer associations** | **Link** |
|  |  | **MCF7-Tamoxifen** | **MCF7- Anastrozole** |  |  |  |
| ***P08603*** | Complement factor H (CFH) | -0.604926922 |  | Modulates complement activation | Secreted. Unfavourable prognostic marker in liver cancer. | https://www.proteinatlas.org/ENSG00000000971-CFH |
| ***P0DOX8*** | Immunoglobulin lambda-1 light chain | -0.602432929 |  | Involved in humoral immunity | Secreted. | Not found in protein atlas |
| ***P10643*** | Complement component C7 (C7) | -0.598843283 |  | Component of the MAC creating pores in target cells | Secreted. Not prognostic in breast cancer but favourable prognosis in liver cancer | https://www.proteinatlas.org/ENSG00000112936-C7 |
| ***P13671*** | Complement component C6 (C6) | -0.725169279 |  | Component of the MAC creating pores in target cells | Secreted. Moderate staining in breast cancer tissue, plasma concentrations similar to that of endometrial and cervical cancer | https://www.proteinatlas.org/ENSG00000039537-C6 |
| ***P15169*** | Carboxypeptidase N catalytic chain (CPN1) | -0.65279501 |  | Offers protection against vasoactive and inflammatory peptides containing C-terminal Arg or Lys | Secreted. Plasma concentrations similar to that of endometrial and cervical cancer | https://www.proteinatlas.org/ENSG00000120054-CPN1 |
| ***P23142*** | Fibulin-1 (FBLN1) | -0.661489817 | -0.62418177 | Incorporated into fibrin-containing ECM fibres, playing a role in cell adhesion and migration | Secreted. Breast cancer tissue shows positivity, not a prognostic marker in breast cancer | https://www.proteinatlas.org/ENSG00000077942-FBLN1 |
| ***P27918*** | Properdin (CFP) | -0.699965214 |  | Positive regulator of the alternate pathway of complement activation | Secreted. Low cancer specificity but associated with innate immune activation | https://www.proteinatlas.org/ENSG00000126759-CFP |
| ***P36980*** | Complement factor H-related protein 2 (CFHR2) | -0.614426301 |  | Complement regulation and a role in lipid metabolism | Secreted. Enriched in liver cancer. | https://www.proteinatlas.org/ENSG00000080910-CFHR2 |
| ***Q12805*** | EGF-containing fibulin-like extracellular matrix protein 1 (EFEMP1) | -0.668481284 | -0.72706493 | Binds EGFR. Role in adhesion & migration. | Secreted. Unfavourable prognostic marker in renal, urothelial and endometrial cancer. Plasma concentrations in breast cancer similar to endometrial cancer. | https://www.proteinatlas.org/ENSG00000115380-EFEMP1 |
| ***P02751*** | Fibronectin 1 (FN1) |  | -1.23479104 | Binds collagen, fibrin, heparin. Involved in cell motility | Secreted factor. Unfavourable prognosis in renal, stomach and urothelial cancer. Positivity in breast cancer stroma | https://www.proteinatlas.org/ENSG00000115414-FN1 |
| ***P02775*** | Platelet basic protein (PPBP) |  | -0.99783108 | Platelet-derived growth factor | Secreted. Prognostic marker in cervical cancer, mean plasma concentration higher in breast cancer | https://www.proteinatlas.org/ENSG00000163736-PPBP |
| ***P04275*** | von Willebrand factor (vWF) |  | -1.53434394 | Promotes platelet adhesion | Secreted. Positivity in breast cancer stroma. Mean plasma concentrations in breast cancer similar to other cancer types | <https://www.proteinatlas.org/ENSG00000110799-VWF> |
| ***P07477*** | Serine protease 1 (PRSS1) |  | -0.92986184 | Activity on peptide linkages involving carboxyl group of lysine /arginine | Secreted. Prognostic marker in endometrial cancer, pancreatic cancer (unfavourable), ovarian cancer (favourable) | <https://www.proteinatlas.org/ENSG00000204983-PRSS1> |
| **Uniprot ID** | **Uniprot Name (gene)** | **Fold Change** | | **Function** | **Human Protein Atlas – cancer associations** | **Link** |
|  |  | **T47D-Tamoxifen** | **T47D-Anastrozole** |  |  |  |
| ***A0A0J9YXX1*** | Immunoglobulin heavy variable 5-10-1 (IGHV5-10-1) | 0.61373 |  | V-segment of variable domain involved in humoral immunity | Secreted. No further pathology or disease data. | https://www.proteinatlas.org/ENSG00000282651-IGHV5-10-1 |
| ***P01011*** | Alpha-1-antichymotrypsin (SERPINA3) | 0.9229 |  | Unclear. Can inhibit neutrophil cathepsin G | Secreted. High expression favourable prognostic marker in breast cancer. | https://www.proteinatlas.org/ENSG00000196136-SERPINA3 |
| ***P01780*** | Immunoglobulin heavy variable 3-7 (IGHV3-7) | 0.699733 |  | V-segment of variable domain involved in humoral immunity | Secreted. Prognostic marker in head and neck cancer (favourable) and renal cancer (unfavourable). | https://www.proteinatlas.org/ENSG00000211938-IGHV3-7 |
| ***P02647*** | Apolipoprotein A-I (APOA1) | 1.104968 |  | Cholesterol transport | Secreted. Prognostic marker in liver cancer (favourable) and renal cancer (unfavourable). Breast stroma shows some positivity and evidence for detection in plasma. | https://www.proteinatlas.org/ENSG00000118137-APOA1 |
| ***P02750*** | Leucine-rich alpha-2-glycoprotein (LRG1) | 0.947613 |  | Mediates cell adhesion via protein-protein interaction and signal transduction | Secreted. Unfavourable prognostic marker in renal cancer and favourable in thyroid cancer. Breast cancer tissue shows weak to moderate staining, with plasma concentrations similar to those found in cervical and endometrial cancer | https://www.proteinatlas.org/ENSG00000171236-LRG1 |
| ***P02790*** | Hemopexin (HPX) | 0.652105 |  | Haem transport | Secreted. Enriched in breast cancer cell lines and glandular tissue, but no prognostic value. Favourable prognostic marker in liver cancer. | https://www.proteinatlas.org/ENSG00000110169-HPX |
| ***P04211*** | Immunoglobulin lambda variable 7-43 (IGLV7-43) | 0.583203 |  | Humoral immunity | Secreted. Prognostic marker in breast cancer (favourable). Weak to moderate staining in breast cancer tissue. | https://www.proteinatlas.org/ENSG00000211652-IGLV7-43 |
| ***P05155*** | Plasma protease C1 inhibitor (SERPING1) | 1.129773 |  | Inhibitor of FXIIa. Regulates coagulation, fibrinolysis and complement activation | Secreted. Favourable prognostic marker in ovarian cancer. Less detected in breast cancer. | https://www.proteinatlas.org/ENSG00000149131-SERPING1 |
| ***P07477*** | Serine protease 1 (PRSS1) | -0.72828 |  | Activity on peptide linkages involving carboxyl group of lysine /arginine | Secreted. Prognostic marker in endometrial cancer, pancreatic cancer (unfavourable), ovarian cancer (favourable) | https://www.proteinatlas.org/ENSG00000204983-PRSS1 |
| ***P23142*** | Fibulin-1 (FBLN1) | -0.75165 |  | Incorporated into fibrin-containing ECM fibres, playing a role in cell adhesion and migration | Secreted. Breast cancer tissue shows positivity, not a prognostic marker in breast cancer | https://www.proteinatlas.org/ENSG00000077942-FBLN1 |
|  | Serum amyloid A-4 protein (SAA4) | 0.9536 |  | Acute phase reactant | Secreted. Enhanced in liver cancer. Negative staining in breast cancer tissue. | https://www.proteinatlas.org/ENSG00000148965-SAA4 |
| ***P35858*** | Insulin-like growth factor-binding protein complex acid labile subunit (IGFALS) | 0.701617 |  | Cell adhesion, protein complex formation | Secreted. Prognostic marker in liver cancer (favourable) and endometrial cancer (unfavourable). Weak staining in breast cancer tissue. | https://www.proteinatlas.org/ENSG00000099769-IGFALS |
| ***P0DOY3*** | Immunoglobulin lambda constant 3 (IGLC3) |  | -2.92957 | Humoral immunity | Secreted. Favourable prognostic marker in breast cancer, low to moderate expression. | https://www.proteinatlas.org/ENSG00000211679-IGLC3 |
